# Supplementary material for: Development of a new genotype–phenotype linked antibody screening system
Source: eLife. 2024 Nov 19;13:RP95346. doi: 10.7554/eLife.95346 (PMC11575895; doi:10.7554/eLife.95346)
Supplement: Supplementary file 1. — (a) Oligonucleotides. (b) Oligonucleotides. [file elife-95346-supp1.docx]

Supplementary Tables

**Supplementary File 1a. Oligonucleotides**

| Primer | Application | Sequence |
| --- | --- | --- |
| First-strand cDNA synthesis | | |
| Oligo(dT)18 | Primer for cDNA synthesis | TTTTTTTTTTTTTTTTTT |
| Second-strand cDNA synthesis | | |
| SMART_dT | TdT oligo | AAGCAGTGGTATCAACGCAGAGTAC(T)_30_VN |
| Mouse IGH/IGK primer | |  |
| TdT_L_BsaI_heavy | mouse IgG heavy-chain cDNA | CTAGGGTCTCAAGCAGTGGTATCAACGCAGAGTAC |
| msVHE |  | GAGGTGCAGCTGCAGGAGTCTGG |
| W-gamma-outer |  | AGAAGGTGTGCACACCGCTGGAC |
| mC_G new2 |  | ATCTCCACACACAGGGGCCAGTGGATAGAC |
| BsaI_IL6sp_L |  | CTAGGGTCTCAAGCAGATGAACTCCTTCTCCACAAGCG |
| mC_G_new2_BsaI |  | TCCTAGGTCTCCCACACACAGGGGCCAGTGGATAGAC |
| TdT_L_BsaI_light | mouse IgK light-chain cDNA | CTAGGGTCTCAGTGGTATCAACGCAGAGTAC |
| mvkappa |  | GAYATTGTGMTSACMCARWCTMCA |
| mCkappa-outer |  | ACTGAGGCACCTCCAGATGTT |
| mC_K_new |  | AGGCACCTCCAGATGTTAACTGCTCACTGG |
| BsaI_IL6sp_kappa_L |  | CTAGGGTCTCAGTGGTATGAACTCCTTCTCCACAAGCG |
| mC_K_new_BsaI |  | TCCTAGGTCTCCTCCAGATGTTAACTGCTCACTGG |

**Supplementary File 1b. Oligonucleotides**

| Primer | Application | Sequence |
| --- | --- | --- |
| Next Generation sequencing | | |
| M13fwd_i5_mC_G_new2^a^ | Well index | TGTAAAACGACGGCCxxxxxxxxATCTCCACACACAGGGGCCAGTGGATAGAC |
| M13fwd_i5_mC_K^a^ |  | TGTAAAACGACGGCCxxxxxxxxGCACCTCCAGATGTTAACTG |
| M13fwd_i5_mC_L^a^ |  | TGTAAAACGACGGCCxxxxxxxxAGCTCCTCAGRGGAAGGTG |
| M13rev_i7_msVHE^b^ |  | GGAAACAGCTATGACyyyyyyyyGAGGTGCAGCTGCAGGAGTCTGG |
| M13rev_ i7_mvkappa^b^ |  | GGAAACAGCTATGACyyyyyyyyGAYATTGTGMTSACMCARWCTMCA |
| i5-Fwd^c^ | Plate index | AATGATACGGCGACCACCGAGATCTACACxxxxxxxx  ACACTCTTTCCCTACACGACGCTCTTCCGATCTTGTAAAACGACGGCC |
| i7-Rev^d^ |  | CAAGCAGAAGACGGCATACGAGATyyyyyyyy  GTGACTGGAGTTCAGACGTGTGCTCTTCCGATCTGGAAACAGCTATGAC |
| i5-Rev^c^ |  | AATGATACGGCGACCACCGAGATCTACACxxxxxxxx  ACACTCTTTCCCTACACGACGCTCTTCCGATCTGGAAACAGCTATGAC |
| i7-Fwd^d^ |  | CAAGCAGAAGACGGCATACGAGATyyyyyyyy  GTGACTGGAGTTCAGACGTGTGCTCTTCCGATCTTGTAAAACGACGGCC |

^a^xxxxxxxx: sequence showing the well index from D501 to D508. ^b^yyyyyyyy: sequence showing the well index from D701 to D712. ^c^xxxxxxxx: sequence showing the plate index from D501 to D508. ^d^yyyyyyyy: sequence showing the plate index from D701 to D712.
